# Supplementary material for: Effectiveness of a multi-country implementation-focused network on quality of care: Delivery of interventions and processes for improved maternal, newborn and child health outcomes
Source: PLOS Glob Public Health. 2024 Mar 4;4(3):e0001751. doi: 10.1371/journal.pgph.0001751 (PMC10911613; doi:10.1371/journal.pgph.0001751)
Supplement: Attachment — Submitted filename: PGPH-D-23-00351 Response to Reviewers .docx [file pgph.0001751.s004.docx]

PGPH-D-23-00351
Effectiveness of a multi-country implementation-focused network on quality of care: delivery of interventions and processes for improved maternal, newborn and child health outcomes
PLOS Global Public Health

Dear Editor,

Thank you for providing feedback on our work and giving our team the opportunity to improve our manuscript. We would like to also thank Dr Salim Masoud for her time reviewing our manuscript.

Please find below our response to the comments received.

Best regards,

Prof. Tim Colbourn and Dr Nehla Djellouli, on behalf of the authors

**Journal Requirements:**

1. Please provide additional details regarding participant consent. In the ethics statement in the Methods and online submission information, please ensure that you have specified what type you obtained (for instance, written or verbal, and if verbal, how it was documented and witnessed). If your study included minors, state whether you obtained consent from parents or guardians. If the need for consent was waived by the ethics committee, please include this information.

Authors response: We have specified in our Methods section that written informed consent was obtained (L. 167). Please note no minors took part in our study.

2. We noticed that you used "unpublished data" in the manuscript. We do not allow these references, as the PLOS data access policy requires that all data be either published with the manuscript or made available in a publicly accessible database. Please amend the supplementary material to include the referenced data or remove the references.

Authors response: We have reviewed our reference list and made the changes as requested. We have included the Medrxiv preprints of the other papers in our QCN Evaluation collection also currently under review with PLOS Global Public Health (references 7, 10, 20, 34). Two of our QCN Evaluation papers (refs 19 and 29) are not yet submitted to PLOS Global Public Health – we could instead cite S1_Text, the overview of our collection of papers, if that works better. We have added references to the Malawi and Uganda quality of care roadmaps (references 30 and 31).

3. We have noticed that you have uploaded Supporting Information files, but you have not included a list of legends. Please add a full list of legends for your Supporting Information files after the references list.

Authors response: The list of legends for the Supporting Information files has now been moved after the references list.

**Reviewers' comments:**

Reviewer's Responses to Questions

Reviewer #1: The manuscript addresses an important component of Quality of care, an aspect to focus towards reducing Maternal, Newborn and Child deaths. Few comments;
1. Justification of choosing the 4 countries out of 11 need to be clear. Narration of the 4 selected counties have been provided but NOT clear why on these four countries only.

Authors response: Thank you for highlighting this issue. We have explained in our methods (L110-111) that the countries were chosen because they represent a range of contexts: “To evaluate the work of the QCN, we conducted multiple embedded case studies[12] in four countries – Bangladesh, Ethiopia, Malawi and Uganda – chosen for the spectrum of contexts they represented.” At the end of the paragraph (L118-119), the reader is invited to consult Supplement 2 for more details on the case study settings and contexts.

We were not able to evaluate all 11 countries due to funding limitations. However, we believe that the case country analyses we conducted as part of this evaluation across those four different contexts have findings relevant for all the QCN countries, and we also evaluated the global level which works across the 11 countries. This has been confirmed during the dissemination of our findings at the last QCN international meeting in March 2023 in Accra, Ghana.

2. The effectiveness: Intervention delivery and process could be affected by the Global set up at the National level withing countries but also the involvement of international partners and other donors with parallel projects in these countries. This could have affected the country ownership and regarded as another project to be learned. This came out very clearly in all the findings of the four countries i.e Low awareness especially at the Sub national level (Meso and Micro levels), Delay or No integration, an extreme example from Ethiopia using parallel system, low implementation at the local level in all 4 countries except for Bangladesh where there was already an existing platform from Nutrition and population, with low accountability. In most of these countries, the learning came from international partner driven efforts like UNICEF, Save the Children and USAID with minimal efforts to call for country partnership, leveraging resources and create Government commitment to allocate specific budget lines for implementation at Sub national levels. YET, the evaluation has not shared any learning from the Global level.
The authors may need to comment on this, so we learn on Global responsibilities in supporting these initiatives and bringing together partners within countries including the National and sub national level. Contextual factors need to be thought and discussed.
Authors response: Global QCN actors supported these initiatives by facilitating the learning across countries. We present in the results sub-section “QCN effectiveness at the global level” (L207-257) ways the global level supported and facilitated the learning. We go in more details on the topic in another paper of our collection (Mwandira et al.) where we discuss the role of the global level in facilitating innovation, learning and sharing. But the QCN was designed for countries’ MoHs to take ownership of the network in-country and be the ones to bring resources and partners together as we explained in our paper (L257-267) and delved into across the first two papers of this collection (Shawar et al., Akter et al.). It was not really the role of global partners to coordinate in-country work at the local levels. Global level responsibilities at this point were only to provide technical support to countries (L208-221).

3. The authors highlighted that the QCN was siloed and disjointed but didn't recommend strongly on what need to be considered as we set up these initiatives at National and local level as well as Global.

Authors response: Thank you for your comment. We have added in our discussion some recommendations around the need for domestic funding and homegrown quality improvement expertise (L.668-671): “Going forward, scale-up and implementation efforts to improve quality of care needs to be done with dedicated budget lines. Ideally, domestic funding would also be directed towards homegrown quality improvement expertise.” We also brought attention to the need for facility- and district-wide systems improvements (L.713-718): “However, meso and macro systems improvements can be much harder to achieve than frontline improvements at learning sites, and this has not been a big focus of the network so far. They are often needed to enable frontline improvements, for example, to improve availability of well trained and motivated staff, ensure timely supplies of drugs and equipment. Embedding quality throughout the system is a longer-term project – so ideally this network needs to run for at least another 5 years to achieve strong results on quality and case fatality rates nationally in each country.”

4. Data from Global view is critical, not sure why excluded in the survey

Authors response: We agree that the data from the global level is critical. The aim of the survey however was to gather the views of national and local QCN actors on QCN implementation in-country so a tailored survey for each country was run for that purpose. As such, global QCN actors were not in a position to answer the questions asked in the survey. However, respondents included implementing partners working directly in the country (e.g. Save the Children, UNICEF, USAID, WHO country offices, etc.).

We have added the aim of the survey to our method section (L123-124): “a quantitative survey to understand QCN actors’ awareness and perceptions of the network in each country” to clarify that the survey was to understand perceptions of the network in each country so therefore the survey does not involve global actors.

For our stakeholder network analysis that we describe in another paper of our collection (Mukinda et al.), we ran a different survey – this time including global actors – to map out interactions amongst global level actors and between global and national level actors.

5. Ownership at the country level - require explanation

Authors response: Ownership is not a focus of this paper (indeed the word is only in the paper twice and not in the context of country level ownership) so we are unsure of what you are referring to here. Ownership of the work of the network by country government ministries of health is a focus of another paper in our QCN evaluation collection (Akter et al) however, and in that paper, we define ownership and evaluate it.

6. PLOS authors have the option to publish the peer review history of their article ([what does this mean?](https://eur01.safelinks.protection.outlook.com/?url=https%3A%2F%2Fjournals.plos.org%2Fglobalpublichealth%2Fs%2Feditorial-and-peer-review-process%23loc-peer-review-history&data=05%7C01%7Cn.djellouli%40ucl.ac.uk%7Cfa14b5e5baf24eb6bc2808db7bcb4ca9%7C1faf88fea9984c5b93c9210a11d9a5c2%7C0%7C0%7C638239885315728584%7CUnknown%7CTWFpbGZsb3d8eyJWIjoiMC4wLjAwMDAiLCJQIjoiV2luMzIiLCJBTiI6Ik1haWwiLCJXVCI6Mn0%3D%7C3000%7C%7C%7C&sdata=XIdK61FfjWK9C6aMu36BVgCU%2B8oJzj4lFSHbmOPjxSc%3D&reserved=0)). If published, this will include your full peer review and any attached files.


**Do you want your identity to be public for this peer review?** If you choose “no”, your identity will remain anonymous but your review may still be made public.


For information about this choice, including consent withdrawal, please see our [Privacy Policy](https://eur01.safelinks.protection.outlook.com/?url=https%3A%2F%2Fwww.plos.org%2Fprivacy-policy&data=05%7C01%7Cn.djellouli%40ucl.ac.uk%7Cfa14b5e5baf24eb6bc2808db7bcb4ca9%7C1faf88fea9984c5b93c9210a11d9a5c2%7C0%7C0%7C638239885315728584%7CUnknown%7CTWFpbGZsb3d8eyJWIjoiMC4wLjAwMDAiLCJQIjoiV2luMzIiLCJBTiI6Ik1haWwiLCJXVCI6Mn0%3D%7C3000%7C%7C%7C&sdata=1s9mNEb1FjQeUt%2F6t3AIIeV%2FDFw1Y8w%2BwZxEeiQv1Bo%3D&reserved=0).

Reviewer #1: **Yes:** NAHYA SALIM MASOUD
