# Supplementary material for: Effectiveness of a multi-country implementation-focused network on quality of care: Delivery of interventions and processes for improved maternal, newborn and child health outcomes
Source: PLOS Glob Public Health. 2024 Mar 4;4(3):e0001751. doi: 10.1371/journal.pgph.0001751 (PMC10911613; doi:10.1371/journal.pgph.0001751)
Supplement: Attachment — Submitted filename: QCN Paper 3 response to additional review.docx [file pgph.0001751.s005.docx]

PGPH-D-23-00351R1
Effectiveness of a multi-country implementation-focused network on quality of care: delivery of interventions and processes for improved maternal, newborn and child health outcomes
PLOS Global Public Health

1^st^ November 2023

Dear Editor,

Thank you for providing feedback from an additional reviewer on our work and giving our team the opportunity to further improve our revised manuscript. Please find below our response to the comments received.

Best regards,

Prof Tim Colbourn and Dr Nehla Djellouli, on behalf of all authors

Journal Requirements:

1. Please review your reference list to ensure that it is complete and correct. If you have cited papers that have been retracted, please include the rationale for doing so in the manuscript text, or remove these references and replace them with relevant current references. Any changes to the reference list should be mentioned in the rebuttal letter that accompanies your revised manuscript. If you need to cite a retracted article, indicate the article’s retracted status in the References list and also include a citation and full reference for the retraction notice.

Authors response: reviewed and correct.

2. We noticed that you used "unpublished" in the manuscript. We do not allow these references, as the PLOS data access policy requires that all data be either published with the manuscript or made available in a publicly accessible database. Please amend the supplementary material to include the referenced data or remove the references.

Authors response: we previously responded to this comment with regard to our other papers that are under-review and are referenced in the paper as pre-prints. Please could you clarify what you are referring to here as we don’t include unpublished data. We do state in the manuscript that we reviewed “unpublished documents” as part of our document review process (lines 162–167): “Finally, we triangulated the data collected with a document review that included all relevant published and unpublished documents and communications relating to QCN at the global, national and district levels in the case study countries. These included strategy and management documents, operational plans, directives, formal minutes, and reports (see [S2_Text]). We were able to access unpublished documents via WHO and Ministry of Health QCN contacts.”. These are mainly strategy and other internal government documents from our case study countries, and are described in S2_Text (section 3.2 and Table 2 of S2_Text). We do not have the authority or permission to make these unpublished documents available. We hope this is ok and is not an issue given we only used these unpublished documents for triangulation. Kindly please also note that S2_Text is common to other papers in this collection, 3 of which have already been published by PLOS Global Health (Tesfa et al 2023, Lemma et al 2023, Mukinda et al 2023).

Reviewer’s comments

Reviewer #1: The authors has addressed all comments as per reviewers recommendations. This is an important information to be shared to the public.

Authors response: Thank you very much for your review and we are glad you recognise the importance of our paper.

Reviewer #2: Thank you for the opportunity to review this revised manuscript (I was not part of the previous round of review), which provides a fascinating account of the work done by a network (QCN) launched in Malawi in 2017. This network embodies the values of ethical global health principles as part of its strategic objectives. This paper is the third account of evaluation of the work done in four of the 11 GCN countries. The study design is that of multiple embedded case studies, reviewing perspectives and data sources across a 6-year period (2016 - 2022). A staggering number of 248 interviews were conducted across a 3 year period, involving key stakeholders across all levels of governance. The authors provided an account of measures implemented to navigate the challenges posed by the Covid pandemic.

Authors response: Thank you for taking the time to review of our paper.

The following aspects could enhance the manuscript further, mainly to ensure complete reporting in line with the COREQ guidelines:
1. Please clarify the background and qualifications of the members of the QCM Evaluation Group who conducted the interviews. What was their relationship with the context and participants?

Authors response: Thanks for these helpful suggestions. We have added a paragraph on reflexivity to the end of our methods section: “QCN Evaluation group members who were from, and based in, the countries being researched, conducted the interviews in each country. They were experienced qualitative researchers familiar with the culture, language, context and health system in the country, though not working in the health system or the particular health facilities involved. As such they were non-participant observers and interviewers.” [this paragraph continues below in response to your two other points]

2. Please clarify the process of "non-participant" observations: how did these members of the Evaluation Group prevent possible Hawthorne effects?

Authors response: [added reflexivity paragraph, continued from above] “We aimed to limit Hawthorne effects – behaviour change in response to being observed – by conducting observations over a number of consecutive days in each selected facility and in a non-obtrusive, non-judgemental way, with our researchers making it clear to those being observed that all data collected would remain anonymous and that there would be no consequences of the observations for those being observed.”

3. It sounds like this was a large group of members in the Evaluation Group - how did you ensure consistency in the fieldwork and minimize any potential impact on validity and trustworthiness? How would you account for the statements of positionality/reflexivity in terms of the relationships with the contexts and participants? It is reassuring to read the considerations around team meetings of coders and how the results of the survey was used to triangulate with the qualitative data.

Authors response: As you note we worked hard to ensure consistency in coding of our qualitative data between team members. We also did a lot of training and re-training of interviewees and coders to ensure consistency and high-quality work. We have added the following sentence on this at line 184: “We worked to ensure consistency in our qualitative data collection and analysis via holding regular interactive group training sessions with all researchers in the team, led by senior members of the team”.

This is in addition to the group coding and other efforts we already mention in the following 10 lines: “Over two years, many of our research team, including nine co-authors for this paper, were involved in coding data in the different case studies. Six of the co-authors were also actively involved in local data collection and familiar with the local context. Regular team meetings and rechecks took place during each round of coding in order to ensure inter-coder consistency and that coders remained close to the theories underpinning the codebook. Team meetings were also an opportunity for coders to put forward new codes they deemed relevant to refine the ‘theory’ codes or to address gaps in ‘case study’ codes. The quantitative data was analysed independently in the first place, by conducting descriptive statistics of respondents and the percentages of respondents giving responses to each question. In [S2_Text], we provide a more detailed explanation of the quantitative data analysis process. After analysis, the results of the survey were triangulated with the qualitative data.”

Please see our response to point 1 above for how we account for the statements of positionality/reflexivity in terms of the relationships with the contexts and participants.
