# Supplementary material for: Effectiveness of a multi-country implementation-focused network on quality of care: Delivery of interventions and processes for improved maternal, newborn and child health outcomes
Source: PLOS Glob Public Health. 2024 Mar 4;4(3):e0001751. doi: 10.1371/journal.pgph.0001751 (PMC10911613; doi:10.1371/journal.pgph.0001751)
Supplement: Attachment — Submitted filename: QCN Paper 3 response to editors additional comments_final.docx [file pgph.0001751.s006.docx]

PGPH-D-23-00351R2
Effectiveness of a multi-country implementation-focused network on quality of care: delivery of interventions and processes for improved maternal, newborn and child health outcomes
PLOS Global Public Health

1^st^ December 2023

Dear Editor,

Thank you for providing additional comments on our paper. Please find below our response to the comments received.

Best regards,

Prof Tim Colbourn, Geremew Gonfa, Asebe Tufa and Dr Nehla Djellouli, on behalf of all authors

Journal Requirements:

1. Please review your reference list to ensure that it is complete and correct. If you have cited papers that have been retracted, please include the rationale for doing so in the manuscript text, or remove these references and replace them with relevant current references. Any changes to the reference list should be mentioned in the rebuttal letter that accompanies your revised manuscript. If you need to cite a retracted article, indicate the article’s retracted status in the References list and also include a citation and full reference for the retraction notice.

Authors response: reviewed and correct. Refs 10 and 20, which are two other papers from our QCN Evaluation collection, have now been published by PLOS Global Public Health, so we have updated these references to reflect this (previously the medrxiv pre-prints were referenced)

Editor’s comments

Thank you for the well-written paper and for making revisions according to the reviewers' comments.

Authors response: Thank you very much.

There are still a few suggestions that I think the paper could benefit from. Please consider the following points and make appropriate changes before I accept the paper for publication.

Authors response: Thank you, please see our responses below.

The findings (in the Results and especially in the Abstract) seem to be quite similar in the four QCN focus countries. Were there any differences between the four countries?

Authors response: In each theme, an attempt was made to present key achievements and limitations in each country (to indicate the similarities and differences in the four countries). For instance, please refer line numbers 416-460, 686-693.

Was there cross-country learning (exchange of knowledge and experience)?

Authors response: Cross-country learning is covered in two other papers in our collection of papers covering the QCN Evaluation. One, which has just been published by Akter et al [Ref 10] “Influences on policy-formulation, decision-making, organisation and management for maternal, newborn and child health in Bangladesh, Ethiopia, Malawi and Uganda: the roles and legitimacy of a multi-country network” (see Table 1 of this paper and associated text): <https://journals.plos.org/globalpublichealth/article?id=10.1371/journal.pgph.0001742>

And another, by Mwandira et al [Ref 29] “Do Networks facilitate innovation, learning and sharing? An evaluation of the Quality-of-Care Network (QCN) in Bangladesh, Ethiopia, Malawi, and Uganda”, which is under review at PLOS Global Public Health (PGPH-D-23-02329)

This is important because the four countries have different health system governance and accountability, which is also reflected in the organisation of maternal and newborn care. For example, how did the governance and accountability system work in Ethiopia, given the highly decentralised health system and the health extension programme? How did it differ from other countries? how did this influence efforts to improve the quality of care? How did other countries learn from it?

Authors response: governance, including in relation to (de)centralisation and the lack of focus of the network (to date) on the periphery of the health system in each country is also covered more extensively in our paper by Akter et al [Ref 10] “Influences on policy-formulation, decision-making, organisation and management for maternal, newborn and child health in Bangladesh, Ethiopia, Malawi and Uganda: the roles and legitimacy of a multi-country network”: <https://journals.plos.org/globalpublichealth/article?id=10.1371/journal.pgph.0001742>

and we wouldn’t want to duplicate material from that paper in this paper.

It is clear that Ethiopia has a decentralized health system with a multi-tier administration system. Ethiopia’s health service is broadly structured into a three-tier system; primary, secondary and tertiary level of care. The primary level of care unit is composed of a primary hospital, health center, and health post. Secondary level health care comprises a general hospital. Tertiary level health care comprises a specialized hospital. The quality of care network initiative was implemented in each level of care and facility types; health centers, general hospitals and referral hospitals. QCN only included government actors at the national, regional and facility level: hospitals and health centers, not health posts – so we didn’t evaluate the contribution of the health extension program at the lowest level (as it was not part of the network activities). But, we thematically discussed various actors at each tier (federal, regional, and facility) including in relation to awareness, engagement, actions and accountability, including the similarities and variations between the four countries. For example, we have mentioned why some regions in Ethiopia have been excluded (Lines 383-384: “Several regions were excluded due to security concerns given the political instability in Ethiopia at the time of selection”). In this multi-tier system we have presented results indicating regional states have financial autonomy, which enable them to divert QCN budget to other issues such as COVID, which affected the effectiveness of QCN implementation (line 345-347).

More points along these lines would make the abstract more attractive. I have seen that most of the answers to these questions are there, but a bit scattered in the paper. It would be great to have a focused reflection to maximise the readership and impact of the paper.

Authors response: Thank you for this helpful suggestion, we have added text to the abstract highlighting similarities and differences between countries – please see tracked changes edits to lines 43-49.
